# Drone and Worker Brood Microclimates Are Regulated Differentially in Honey Bees, *Apis mellifera*

**DOI:** 10.1371/journal.pone.0148740

**Published:** 2016-02-16

**Authors:** Zhiyong Li, Zachary Y. Huang, Dhruv B. Sharma, Yunbo Xue, Zhi Wang, Bingzhong Ren

**Affiliations:** 1 Jilin Key Laboratory of Animal Resource Conservation and Utilization, School of Life Sciences, Northeast Normal University, Changchun, Jilin Province, China; 2 Jilin Institute of Apicultural Research, Jilin, Jilin Province, China; 3 Department of Entomology, Michigan State University, East Lansing, MI 48824, United States of America; 4 Center for Statistical Training and Consulting, Michigan State University, East Lansing, MI 48824, United States of America; Colorado State University, UNITED STATES

## Abstract

**Background:**

Honey bee (*Apis mellifera*) drones and workers show differences in morphology, physiology, and behavior. Because the functions of drones are more related to colony reproduction, and those of workers relate to both survival and reproduction, we hypothesize that the microclimate for worker brood is more precisely regulated than that of drone brood.

**Methodology/Principal Findings:**

We assessed temperature and relative humidity (RH) inside honey bee colonies for both drone and worker brood throughout the three-stage development period, using digital HOBO^®^ Data Loggers. The major findings of this study are that 1) both drone and worker castes show the highest temperature for eggs, followed by larvae and then pupae; 2) temperature in drones are maintained at higher precision (smaller variance) in drone eggs and larvae, but at a lower precision in pupae than the corresponding stages of workers; 3) RH regulation showed higher variance in drone than workers across all brood stages; and 4) RH regulation seems largely due to regulation by workers, as the contribution from empty honey combs are much smaller compared to that from adult workers.

**Conclusions/Significance:**

We conclude that honey bee colonies maintain both temperature and humidity actively; that the microclimate for sealed drone brood is less precisely regulated than worker brood; and that combs with honey contribute very little to the increase of RH in honey bee colonies. These findings increase our understanding of microclimate regulation in honey bees and may have implications for beekeeping practices.

## Introduction

Ambient environmental conditions fluctuate widely due to day/night and change of seasons. Yet many social insects are able to regulate environmental conditions, such as temperature (T), relative humidity (RH), and carbon dioxide levels within their nests [1, 2]. Colonies of western honey bee, *Apis mellifera*, maintain their brood nest temperature around 34–36°C, which is optimal for brood development [3–5]. The stable temperature is maintained by honey bees through various control mechanisms. Honey bees increase colony temperature by isometric contraction of thoracic muscles to produce heat [6]. Workers increase heat transfer efficiency by pressing their heated thoraces against the caps and walls of brood cells [7, 8]. The heating is performed by young bees (nurses) who have higher thoracic temperatures [9]. Besides providing optimal temperature for brood development, elevated temperatures can also defend against fungal infections [10] and varroa mites [11]. But temperatures above 36°C for extended times are harmful to the brood and may result in developmental abnormalities or death [12, 13]. To decrease temperature, workers fan their wings to cool the colony [14], and at the same time spread water or diluted nectar to induce evaporative cooling [15]. Honey bee workers can also shield the comb from external heat sources to prevent brood from overheating [16].

Drones and workers have different roles in a colony. This is reflected in many physiological, morphological and behavioral differences [17]. Workers perform many different tasks, yet the only function of drones is to produce sperm and mate with a queen [18]. Drone production thus is regulated and not produced all the time [19, 20]. Drones are also more costly to produce compared to workers due to their larger size [17, 21, 22]. Because the functions of drones are more related to colony reproduction, and those of workers relate to both colony survival and reproduction, we hypothesized that the environment for worker brood is more precisely regulated than that of drone brood.

Thermoregulation has been the most extensively studied aspect of nest homeostasis [23]. Different stages of brood may have different optimal temperatures. Büdel [24] first noticed that worker pupae had higher temperatures than either eggs or larvae. There might also be caste-specific differences in temperature and/or humidity requirement by worker and drone brood. Levin and Collison [25] determined that worker brood is maintained at a significantly higher temperature than drone brood, when both are in the central brood nest of frame, but this difference is not maintained when brood is placed in the outer brood nest region.

In contrast, humidity regulation in honey bee colonies is only sparsely studied [26–28]. There is a controversy whether RH in a bee colony is actively regulated: some thought that humidity inside colonies varies passively [29, 30], while Ellis *et al*. [31] concluded that humidity in colonies is actively controlled by workers. Human *et al*. [27] indicated workers can only control humidity in the colony within sub-optimal limits. Brood comb has been shown to be able to function as a humidity buffer in nests [28].

In this study we measured temperature and humidity in both worker and drone brood, including the developmental stages of eggs, larvae, and pupae. We intended to test four different hypotheses. 1). Are there different temperature and humidity requirements among the different development stages, namely, eggs, larvae and pupae? 2). Are there differences in temperature and humidity requirements for worker and drone brood? 3). Are worker brood temperature and humidity regulated more precisely than that of drone brood? And 4). Do honey combs act as a buffer for humidity regulation?

## Materials and Methods

### Honey bees

In May and June 2012, six honey bee colonies (*A*. *mellifera*), located at the Jilin Institute of Apicultural Research (GPS coordinates: 43.72° N, 126.66° E), were used for Experiment I in this study. Bees were housed in Langstroth hives, each with six frames of bees. Another nine 6-frame *A*. *mellifera* colonies were used for Experiment II in the same apiary during April 2013.

### Experiment I. Differences of T and RH in drone and worker brood across three developmental stages

The aim of the experiment is to determine if there are differences across the eggs, larvae and pupae within each caste (worker or drone brood) and also if there are differences between worker and drone brood.

Six colonies were randomly divided into two groups, one group with brood and one broodless. In the brood-right colonies, six frames were arranged as food, worker, worker, drone, drone and food. The worker and drone frames were eggs (~1,700) laid within 24 hours, from 4 different strong source colonies. All the frames were located in the central part of the 10-frame hive box; each side of the six frames group ended with wooden “following boards” (shaped like a frame) to help bees maintain their cluster as is commonly practiced in China. The queen was caged between the two center-most frames (one worker and one drone frame) during the whole experiment. For broodless colonies, six frames with honey, pollen and empty cells were used with the queen caged between the two central frames.

T and RH were measured to the nearest 0.01°C or 0.01% RH using HOBO^®^ T/RH Data Logger-U10-003 (USA, Onset Computer Corporation) which were factory calibrated. To reduce the volume of the instrument (59×44×19mm), the plastic enclosure was removed and the circuit board (54×39×10mm) was put into a plastic bag which had eighteen small holes distributed on both sides. One logger was put between two drone brood frames and one between two worker brood frames to record T and RH, both loggers were at the center of the frames. For T and RH of broodless colonies, one logger was placed between the 2^nd^ and 3^rd^ frame. Ambient T and RH were provided by a logger installed in an instrument shelter in the apiary. All HOBO^®^ loggers were programmed to sample at 30 minutes intervals. Ten loggers were used simultaneously (2 × 3 for worker and drone brood, 3 for broodless colonies, and 1 for ambient).

In this experiment, 3 colonies of bees (equipped with sensors to measure relative humidity and temperature every 30 minutes) were considered with 2 castes of bees (drone and worker), studied through 3 stages of development (egg, larva and pupa).

### Experiment 2: Effect of honey combs on regulation of humidity

The aim of the experiment is to study the effect of honey comb on the regulation of relative humidity. The experiment consisted of three treatments, with “Box-only” (no comb and no bees), “Box+frames” (6 honey frames with honey, pollen and empty cells located in the middle part of each hive, no bees) and “Broodless” (six frames with honey, pollen, and empty cells, plus approximately 15,000 workers and one queen caged between the two central frames). Each treatment consisted of 3 hives. The experiment was conducted over a span of 5 days with measures being taken every 30 minutes.

### Data conversion and statistics

Because RH increases as temperature decreases, even when there is no change in the actual moisture, we standardized RH at a constant 35°C. Mathematically this is similar to using absolute humidity as was done in [27]. The calculations for standardized RH (sRH) were as follows:

$$\text{sRH}={\text{p}}_{\text{ws}}\;*\;\text{RH}\;/\;5560.94,$$

where 5560.94 is the saturation pressure (Pa) of water vapor at 35°C.

p_ws_ at each temperature was calculated as

pws=exp(77.3450+0.0057*T−7235/T)/T^8.2

For Experiment 1, a repeated measures nested analysis of variance (ANOVA) design was implemented separately for each of the 2 outcomes measures (relative humidity and temperature) [32]. Since colonies were intrinsically different, the effect of colony is considered a fixed effect, with the caste (worker or drone) being a nested effect within colony. Finally, stage of development is nested within both stage and colony. The egg stage was measured for 3 days, followed by 6 days of larva and 12 days of pupa development. We assume that the repeated measures of the outcomes taken every 30 minutes were homogenous within each day, and model this with a compound symmetric repeated measures structure. This design was implemented in SAS version 9.4 (SAS Institute, Cary NC).

For Experiment 2, a repeated measures ANOVA design was implemented, with the treatment considered to have a fixed effect [32]. We assume that the repeated measures of the outcome taken every 30 minutes were homogenous within each day, and model this with a compound symmetric repeated measures structure.

## Results

### Experiment I. Differences of T and RH in drone and worker brood across three developmental stages

#### Experiment 1: Temperature

**Fig 1:**
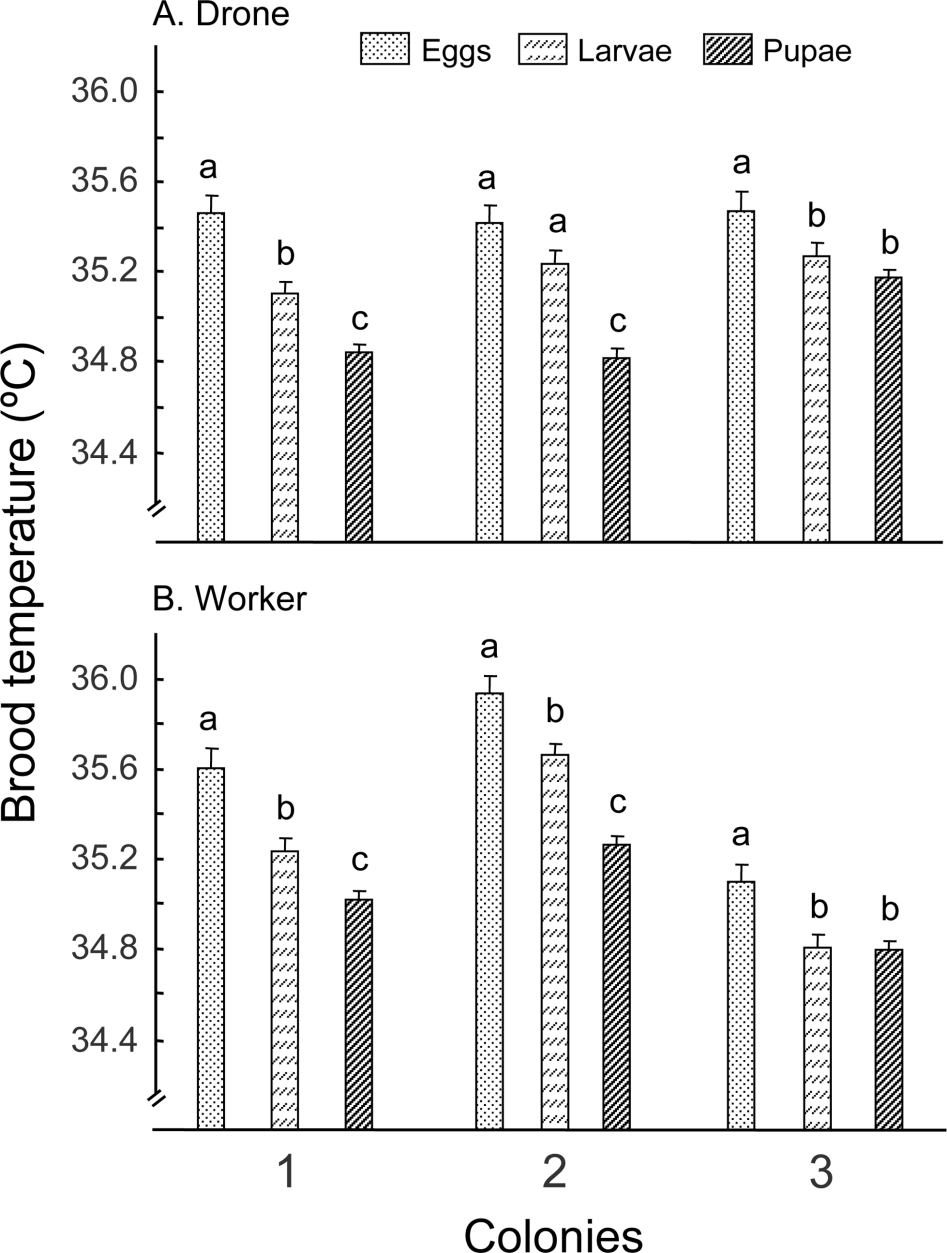
**Temperature of drone (A) and worker (B) brood during eggs, larvae and pupae stages.** Bars with different letters on top of them indicate significant differences (P<0.05) within each colony by Least Square Means after analysis of variance showed a significant effect.

Colony, caste nested within colony and stage nested within caste and colony, all had a significant effect on temperature (Fig 1, Table 1). Due to a significant colony effect, suggesting that each colony behaved differently, we could not make generalizations about differences between castes or among the three stages of each caste, across all three colonies. Instead, we made all preplanned comparisons between worker and drones across each brood stage inside each colony. For different brood stages, the general trend is for both drones and workers to show brood temperature as eggs > larvae > pupae, but this was resolved only in colony 1 (both drone and worker) and colony 2 (worker only). Other data showed at least eggs with a higher temperature than pupae (drone: colony 2 and 3, worker: colony 3, Fig 1, S3 Table). Worker brood always showed a different temperature than drone brood, regardless of stages, across all three colonies. However, drones had lower temperatures than workers in their eggs, larvae, and pupae stages in colony 1 and 2 but this trend is reversed in colony 3, with drones showing higher temperatures across all three stages (S3 Table).

**Table 1:** Tests of fixed effects for honeybee brood temperature.

Effect	Num DF	Den DF	F Value	Pr > F
Colony	2	40	421.80	< .0001
Caste	3	48	680.96	< .0001
Stage	12	48	47.01	< .0001

#### Experiment 1: Relative Humidity

**Fig 2:**
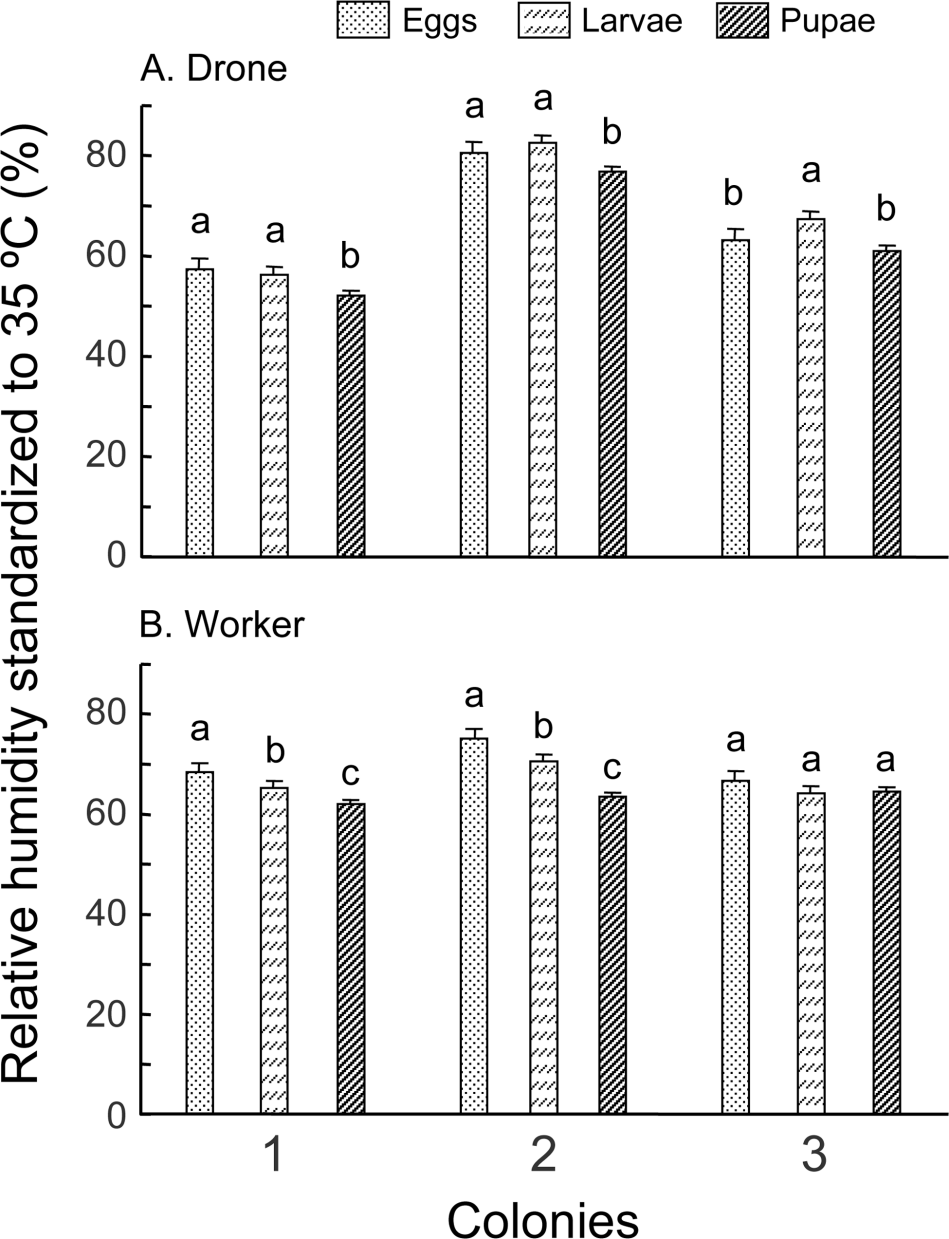
**sRH of drone (A) and worker (B) brood during eggs, larvae and pupae stages.** Bars with different letters on top of them indicate significant differences (P<0.05) within each colony by Least Square Means after analysis of variance showed a significant effect.

Colony, caste nested within colony and stage nested within caste and colony, all had a significant effect on relative humidity (Fig 2, Table 2). Due to a significant colony effect, suggesting that each colony behaved differently, we could not make generalizations about differences between castes or among the three stages of each caste, across all three colonies. Instead, we made all preplanned comparisons between worker and drones across each brood stage inside each colony. sRH differences among the three brood stages in colony 1 and 2 were consistent across both castes, but colony 3 behaved slightly differently. For drone brood, pupae showed a lower sRH than eggs or larvae, except in Colony 3, where pupae showed the same sRH as eggs but lower sRH than larvae. For worker brood, sRH were eggs > larvae > pupae for Colonies 1 and 2 but the three stages remained the same in Colony 3 (Fig 2). Worker brood always showed a different sRH than drone brood, regardless of stages, across all three colonies (S6 Table). However, there is no general trend as to which one is higher. For example, sRH of drone eggs, larvae and pupae were all significantly lower than worker counterparts in Colony 1; but in Colony 2, this is reversed, with drone brood sRH being higher than worker brood in all three stages. In colony 3, drone eggs and pupae were lower but drone larvae were higher in their sRH compared to that of workers (S6 Table).

**Table 2:** Tests of fixed effects for honeybee brood sRH.

Effect	Num DF	Den DF	F Value	Pr > F
Colony	2	40	3279.76	< .0001
Caste	3	48	1003.2	< .0001
Stage	12	48	46.77	< .0001

#### Experiment 1: Variability of T and sRH

We determined the variation of both T and sRH by calculating their variance (VAR) and then F values of various pairwise comparisons. For T, the general trend of VAR was broodless colonies > worker brood > drone brood (Table 3). The only exception is that worker pupae showed a lower variability than drone pupae (F = 1.29, P < 0.05). For sRH, the general trend of VAR was drone brood > broodless colonies = worker brood (Table 4). The only exception was that during pupae stage, worker brood VAR of sRH was lower than broodless colonies.

**Table 3:** Variance of temperature in different developmental stages of workers and drones. Numbers in parenthesis indicate sample size. Numbers in [] denote the ratio was reversed (e.g. F_Worker brood-Drone brood,_ for pupae stage, [1.29] denotes the original F value was 1/1.29).

	Eggs	Larvae	Pupae
Worker brood	0.18 (287)	0.23 (863)	0.09 (1727)
Drone brood	0.04 (287)	0.07(935)	0.12 (2087)
Broodless	0.63 (287)	2.82 (935)	3.84 (2087)
Ambient	17.52 (95)	16.21 (287)	17.55 (575)
F_Worker brood-Drone brood_	4.40*	3.38*	[1.29]*
F_Drone brood-Broodless_	15.75*	41.41*	33.12*
F_Drone brood-Ambient_	438.0*	231.57*	146.25*
F_Broodless-Ambient_	27.81*	5.75*	4.57*

* indicates significant difference (P<0.05) of the F value.

**Table 4:** Variance of relative humidity in different developmental stages of workers and drones. Numbers in parenthesis indicate sample size. Numbers in [] denote the ratio was reversed (e.g. F_Worker brood-Drone brood,_ for pupae stage, [4.53] denotes the original F value was 1/4.53).

	Eggs	Larvae	Pupae
Worker brood	0.42 (287)	0.46 (863)	0.30 (1727)
Drone brood	1.06 (287)	1.32 (935)	1.36 (2087)
Broodless	0.46 (287)	0.50 (935)	0.79 (2087)
Ambient	0.05 (95)	0.13 (287)	0.18 (575)
F_Worker brood-Drone brood_	[2.51]*	[2.86]*	[4.53]*
F_Worker brood-Broodless_	1.09 ns	1.09 ns	2.62*
F_Drone brood-Broodless_	2.29*	2.62*	1.73*
F_Drone brood-Ambient_	21.56*	9.56*	7.37*
F_Broodless-Ambient_	9.40*	3.80*	4.27*

* indicates significant difference (P<0.05) of the F value. ns: not significant.

### Experiment II. Contribution of honey combs to RH regulation

**Fig 3:**
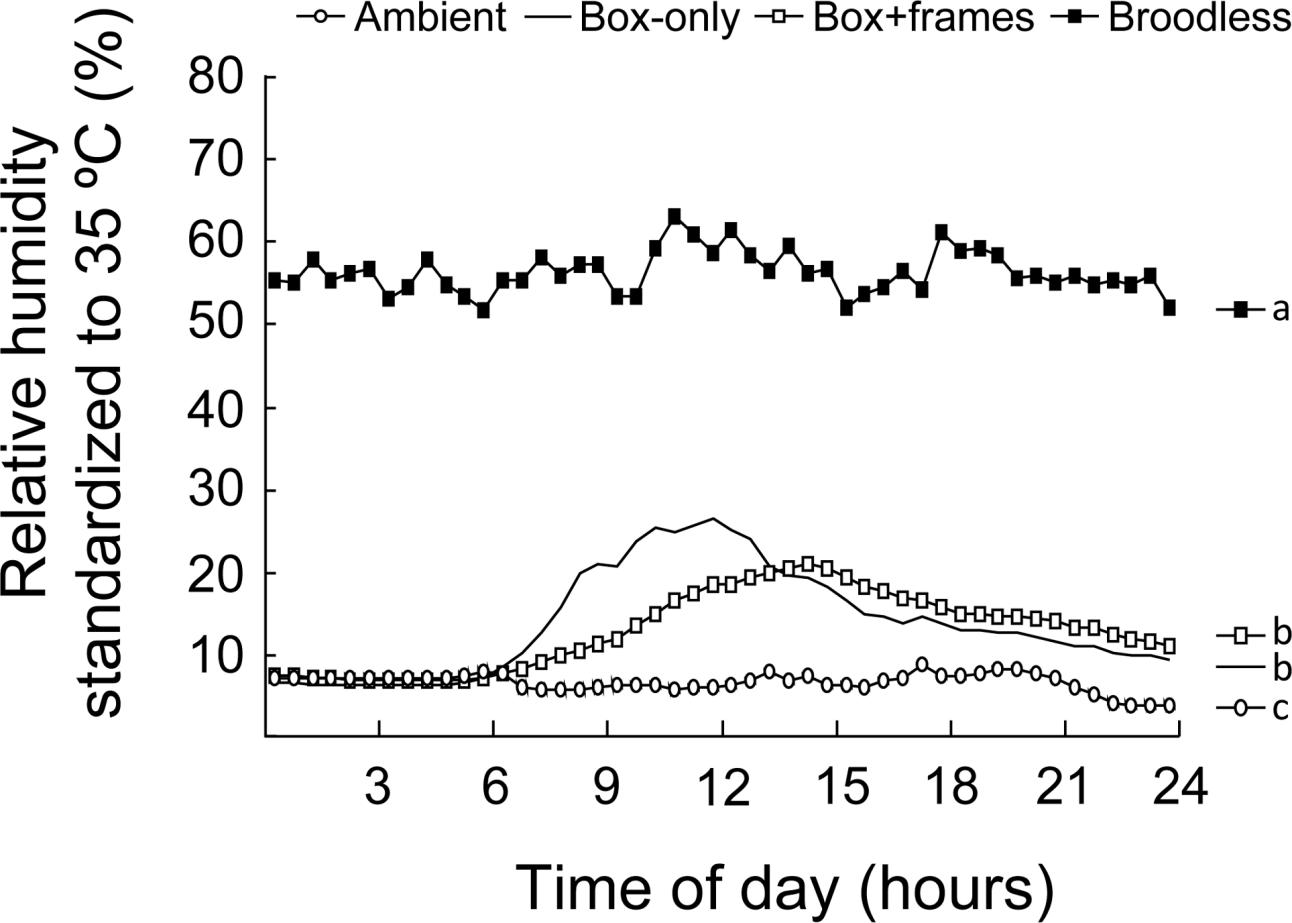
Changes in sRH inside hive boxes when they are Box-only, Box+frames, or Broodless. Data presented as average of three colonies, during one 24-hour period, the third day of experiment. All other days followed the same pattern. Box-only: no frames nor bees; Box+frames: six honey frames with honey, pollen and empty cells located at the middle part of each hive; Broodless: six honey frames and 15,000 workers and one caged queen; Ambient: relative humidity measured in a standard instrument shelter box. Different letters on the right side of the mean indicate the significant differences (P<0.05) by Least Square Means.

In this experiment, the treatments had a significant effect on sRH (Fig 3). Specifically, broodless sRH was significantly higher from all other treatments; ambient sRH was significantly lower than all others, but box+frames were not different from box-only treatments.

## Discussion

The major findings of this study are that 1) both drone and worker castes show the highest temperature for eggs, followed by larvae and pupae (Fig 1); 2) temperature in drones are maintained at higher precision (smaller variance) in drone eggs and larvae, but at a lower precision in pupae than the corresponding stages of workers (Table 3); 3) RH regulation showed higher variance in drone than workers across all brood stages (Table 4); and 4) RH regulation seems largely due to regulation by workers, as the contribution from empty honey combs are much smaller compared to adult workers (sRH _Box+frames_ << sRH _Broodless_) (Fig 3).

We used a standardized RH assuming a constant of 35°C to remove the effects a changing temperature on RH, because the normal inverse relationship between the two will create misleading results without this standardization. Statistically this is equivalent of using absolute humidity, as it was done in another study [27]. However, the advantage of using this “standardized RH” (instead of absolute humidity) is that we have a better “feel” of the humidity levels. Instead of something like 0.08% of absolute humidity, we have a familiar range of RH (e.g. 30–80%) when temperature is fixed at the brood nest temperature at 35°C.

### Pupae required the lowest temperature in both worker and drone brood

Different stages of brood may have different optimal temperatures. Our study showed that in both worker and drone brood, eggs had the highest temperature, followed by larvae and pupae (Fig 1). This is not consistent with previous finding that pupae showing higher temperatures than either eggs or larvae [24]. It is possible that our measuring device is more sensitive and records more accurately than the technology used in the 50s. However higher temperature is known to inhibit fungal pathogens such as chalkbrood [33], which typically attack brood at the larval stage or newly capped brood. More studies are needed to see how robust this pattern is, since we only used three colonies.

### Brood temperatures are different between worker and drone brood

We found that there are temperatures differences across all three development stages, with worker brood temperature slightly higher than drone brood (Colonies 1 and 2, Fig 1). These data are consistent with the findings of Levin and Collison [24]. They concluded that worker brood is maintained at a significantly higher temperature than drone brood in the center of brood nest, but this difference is not maintained in the outer brood nest regions. In our study both worker brood and drone brood were placed near the center in a symmetrical way, and we observed such a difference in 2 out of 3 colonies.

### Caste difference in RH regulation show a different pattern compared to temperature regulation

Our hypothesis was that microenvironment for worker brood might be more tightly regulated than for drone brood. This is not totally supported by our data. For temperature, both eggs and larvae of drones show a lower variance than that of workers, but this pattern is reversed in pupae (Table 3). However, for sRH, drone brood showed a higher variance than worker brood at all three stages (eggs, larvae and pupae, Table 4). sRH in drone brood therefore is not regulated as precisely as that in worker brood. It is not clear which one is more important for brood development, T or sRH. It is possible that sRH regulation might be more costly (requires the foraging for water, for example) compared to T which only consumes more energy. If this assumption is true, then our hypothesis was supported for better humidity control in worker brood compared to drone brood. In other words, bees might regulate humidity more tightly for worker brood, which is important for both survival and reproduction. However, the fact that broodless colonies show similar variance to worker eggs and larvae suggest tight regulation of humidity might not be important for eggs and larvae.

### Honey combs contribute little to humidity regulation

We originally hypothesized that honey comb should be able to passively regulate RH due to honey’s hygroscopic properties. Theoretically, it is possible that combs with honey will be able to absorb moisture when ambient RH is high and then release the moisture when RH is low. However, Box+frames (these frames had honey, but no bees) had 11.70% sRH, which is not significantly higher than the 11.25% for Box-only; while ambient had sRH of 8.18%. We interpret that a single hive body contributed as much moisture as the box with frames (~3%). RH started to climb around 6:30 am in the Box-only and 7:00 am in the Box+frames, with Box+frames showing a delayed climb (max near 15 hrs, instead of at 12 noon for Box-only) as well as more attenuated peak (20.90% max instead of 26.49% max) (Fig 3).

### Humidity in colony is largely due to regulation by honey bee adult workers

RH regulation by workers seems largely due to active regulation, as the contributions from hive box (3%) and empty honey combs (0.45%) are much smaller compared to those from adult workers (55.08% - 11.70% = 43.38% due to worker effort) (Fig 3). Prior to our study, whether RH in a bee colony was actively regulated remained controversial: some thought that humidity inside colonies varies passively [29, 30], while Ellis *et al*. [31] concluded that humidity in colonies is actively controlled by workers. Human *et al*. [27] indicated workers can only control humidity in the hive within sub-optimal limits. Based on our Experiment II, honey bee adult workers not only actively regulated humidity in the bee hive, but also played the most important role in the increase of sRH in the hive (11.70 ± 0.13% and 55.08 ± 0.19% for Box+frames and Broodless respectively, P<0.01).

The mechanisms of microclimate regulation inside a honey bee colony are complex [34, 35]. Our study shows that drone brood might be treated differently from worker brood inside a colony. This makes ecological sense because not only are there physiological differences between the two castes, but the two castes also play different roles inside a colony. Drones are perhaps more “disposable” because they are not needed for survival, only for reproduction. Our data for less tight RH regulation for drone brood supports this hypothesis.

## Supporting Information

S1 Table Least squares means for honeybee brood temperature. (XLS)

S2 Table Stage temperature differences of least squares means within caste and colony of honeybee brood. (XLS)

S3 Table Least squares means for honeybee brood sRH. (XLS)

S4 Table Stage sRH differences of least squares means within caste and colony of honeybee brood. (XLS)

S5 Table Tests of fixed effects for honeybee brood sRH in contribution test of honey combs to RH regulation. (XLS)

S6 Table Least squares means for honeybee brood sRH in contribution test of honey combs to RH regulation. (XLS)

S7 Table sRH differences of least squares means between treatments in contribution test of honey combs to RH regulation. (XLS)
